# An Enhanced Histopathology Analysis: An AI-Based System for Multiclass Grading of Oral Squamous Cell Carcinoma and Segmenting of Epithelial and Stromal Tissue

**DOI:** 10.3390/cancers13081784

**Published:** 2021-04-08

**Authors:** Jelena Musulin, Daniel Štifanić, Ana Zulijani, Tomislav Ćabov, Andrea Dekanić, Zlatan Car

**Affiliations:** 1 Faculty of Engineering, University of Rijeka, Vukovarska 58, 51000 Rijeka, Croatia; jmusulin@riteh.hr (J.M.); car@riteh.hr (Z.C.); 2 Department of Oral Surgery, Clinical Hospital Center Rijeka, Krešimirova Ul. 40, 51000 Rijeka, Croatia; ana.zulijani@sz.uniri.hr; 3 Faculty of Dental Medicine, University of Rijeka, Krešimirova Ul. 40, 51000 Rijeka, Croatia; 4 Department of Pathology and Cytology, Clinical Hospital Center Rijeka, Krešimirova Ul. 42, 51000 Rijeka, Croatia; andrea.dekanic@medri.uniri.hr; 5 Faculty of Medicine, University of Rijeka, Ul. Braće Branchetta 20/1, 51000 Rijeka, Croatia

**Keywords:** AI-based system, data preprocessing, histopathological images, oral squamous cell carcinoma

## Abstract

**Simple Summary:**

An established dataset of histopathology images obtained by biopsy and reviewed by two pathologists is used to create a two-stage oral squamous cell carcinoma diagnostic AI-based system. In the first stage, automated multiclass grading of OSCC is performed to improve the objectivity and reproducibility of histopathological examination. Furthermore, in the second stage, semantic segmentation of OSCC on epithelial and stromal tissue is performed in order to assist the clinician in discovering new informative features. Proposed AI-system based on deep convolutional neural networks and preprocessing methods achieved satisfactory results in terms of multiclass grading and segmenting. This research is the first step in analysing the tumor microenvironment, i.e., tumor-stroma ratio and segmentation of the microenvironment cells.

**Abstract:**

Oral squamous cell carcinoma is most frequent histological neoplasm of head and neck cancers, and although it is localized in a region that is accessible to see and can be detected very early, this usually does not occur. The standard procedure for the diagnosis of oral cancer is based on histopathological examination, however, the main problem in this kind of procedure is tumor heterogeneity where a subjective component of the examination could directly impact patient-specific treatment intervention. For this reason, artificial intelligence (AI) algorithms are widely used as computational aid in the diagnosis for classification and segmentation of tumors, in order to reduce inter- and intra-observer variability. In this research, a two-stage AI-based system for automatic multiclass grading (the first stage) and segmentation of the epithelial and stromal tissue (the second stage) from oral histopathological images is proposed in order to assist the clinician in oral squamous cell carcinoma diagnosis. The integration of Xception and SWT resulted in the highest classification value of 0.963 (σ = 0.042) AUCmacro and 0.966 (σ = 0.027) AUCmicro while using DeepLabv3+ along with Xception_65 as backbone and data preprocessing, semantic segmentation prediction resulted in 0.878 (σ = 0.027) mIOU and 0.955 (σ = 0.014) F1 score. Obtained results reveal that the proposed AI-based system has great potential in the diagnosis of OSCC.

## 1. Introduction

Cancer is a major public health problem and the second leading cause of death in the developed world. Oral cancer (OC; Supplementary Materials Table S1 lists descriptions of the abbreviations and acronyms used) is among the ten most common cancers in Europe and the United States, where more than 90% are squamous cell carcinomas [1,2]. According to the GLOBOCAN database, there were an estimated 377,713 new cases diagnosed, and 177,757 deaths were recorded in 2020 [3]. Despite diagnostic and therapeutic development in OC patients’, mortality and morbidity rates remain high with no advancement in the last 50 years, largely due to late-stage diagnosis when tumor metastasis has occurred [4]. Often, oral squamous cell carcinoma (OSCC) arises from pre-existing lesions of oral mucosa with increased risk for malignant transformation in cancer. Diagnosis and management at the “precancerous” stage and early detection of OSCC improves survival rates and morbidity accompanying the treatment of OSCC [5]. Typically, OSCC is treated primarily by surgical resection with or without adjuvant radiation, which has a major impact on patient quality of life [6]. Until now great strides have been made in understanding the complex process in carcinogenesis, but no reliable tool for prognostic prediction has been found. The tumor-node-metastasis (TNM) staging is widely used in the prognosis, treatment plan, and prediction outcomes of oral cancer in patients with OSCC. However, the inability to incorporate clinical characteristics and individual characteristics of the patient such as lifestyle behaviours reflects on the limitation of the TNM staging in prognostic prediction [7]. Currently, standard methods for detection of oral cancer and the gold standard are clinical examination, conventional oral examination (COE), and histopathological evaluation following biopsy, which can detect cancer in the stage of established lesions with significant malignant changes [8]. However, the main problem in using histopathological examination for tumor differentiation, and like prognostic factor, is the subjective component of the examination, respectively inter- and intra-observer variability [9]. Improving objectivity and reproducibility, respectively reducing inter- and intra-observer variability using Artificial Intelligence (AI) algorithms could directly impact patient-specific treatment intervention by identifying patients’ outcomes. Furthermore, it could assist the pathologist in terms of reducing the load of manual inspections as well as making fast decisions with higher precision.

Recently, many AI algorithms have been proven successful in medical image analysis [10,11,12,13] as well as other various fields of science and technology [14,15,16]. Medical image analysis is one of the areas where histological tissue patterns are used with computer-aided image analysis to facilitate detection and classification of disease [17]. Numerous studies describe the successful implementation of AI algorithms for the detection, staging, and prognosis of all types of cancer. Based on histopathological images Sharma and Mehra proposed a model for automatic multiclass classification of breast cancer using a deep learning approach. Among the different combinations of classifiers VGG16, a convolutional neural network (CNN) architecture which consists of 16 layers that have weights, in a combination with support-vector machine (SVM) classifier resulted in the highest micro- and macro-average (0.93) [18]. Wu et al. used a deep CNN framework in order to predict the risk of lung cancer. CNNs are considered as the most popular deep learning approach in terms of analysing visual imagery. In their study, authors presented that features extracted from histopathological images can be used for the prediction of lung cancer recurrence [19]. By analysing histopathological images Tabibu et al. presented how deep learning algorithms can be used for pan-renal cell carcinoma classification as well as prediction of survival outcome. After breaking the multiclass classification task into multiple binary tasks, deep CNN in a combination with directed acyclic graph (DAG) and SVM proved to be the best performing method and resulted in 0.93 slide-wise AUC [20].

In this research, a two-stage AI-based system for automated multiclass classification of OSCC into three classes, in the first stage, and semantic segmentation of the epithelial and stromal tissue in the second stage is proposed.

The main contributions in this research are as follows:

- The first stage of an AI-based system for multiclass grading of OSCC which can potentially improve objectivity and reproducibility of histopathological examination, as well as reduce the time necessary for pathological inspections.
- The second stage of an AI-based system for segmentation of tumor on epithelial and stromal regions which can assist the clinician in discovering new informative features. It has great potential in the quantification of qualitative clinic-pathological features in order to predict tumor invasion and metastasis.
- A new preprocessing methodology based on the stationary wavelet transform (SWT) is proposed to enhance high-frequency components in the case of multiclass classification and to extract low-level features in the case of semantic segmentation. This approach allows more effective predictions and improves the robustness of the entire AI-based system.

### Related Work

Many studies have used various models, techniques, and methodologies in order to develop AI-solutions for classification and segmentation of oral cancer.

Ariji et al. demonstrated the use of artificial intelligence for the diagnosis of lymph node metastasis in patients with OSCC. The dataset consisted of 441 computed tomography images in total. The performance of the proposed system resulted in 75.4% sensitivity, 81% specificity, and 78.2% accuracy. The obtained results were similar to those found by the radiologists which prove that the proposed AI system can be valuable for diagnostic support [21]. Halicek et al. presented deep learning methods for detection of OSCC. Their dataset was collected from Emory University Hospital Midtown and consisted of 293 tissue specimens. The tissue specimens were imaged with reflectance-based HSI and autofluorescence imaging. With HSI-based methods, OSCC detection may be obtained in less than 2 min with AUC upwards of 0.80 to 0.90 [22]. Horie et al. used CNN to diagnose outcomes of esophageal cancer. The dataset was collected at the Cancer Institute Hospital (Toyko, Japan) and consisted of 8428 training images and 1118 test images. The proposed method could detect oral esophageal cancer with a sensitivity of 98% and distinguish esophageal cancer from advanced cancer with an accuracy of 98% [23]. Tamasiro et al. showed the diagnostic ability of an AI-based system for the detection of oral pharyngeal cancer. The dataset consists of 5400 training images and 1912 validation images, obtained from the Cancer Institute Hospital. CNNs detected pharyngeal cancer with high sensitivity (85.6%) [24]. Jeyaraj et al. used a partitioned deep CNN to detect oral cancer in the early stages. The performance of this partitioned deep CNN resulted in 94% sensitivity, 91% specificity, and 91.4% accuracy. After comparison with other medical image classification algorithms, from obtained results, it can be concluded that the quality of diagnosis was increased [25]. Bhandari et al. proposed a system that consists of a CNN with a modified loss function to minimize the error in predicting and classifying oral cancer. Dataset contains magnetic resonance imaging (MRI) scans which were used for training and testing the proposed system. Based on the results, the proposed system achieved an accuracy of 96.5% [26]. In order to detect oral cancer in the early stages, Xu et al. established a three-dimensional CNN algorithm. Their results show that 3DCNNs outperform 2DCNNs in identifying benign and malignant lesions [27]. Welikala et al. presented the automated detection and classification of oral cancer in the early stages. Images were gathered from clinical experts as a part of the MeMoSA project. Image classification algorithm based on deep CNN resulted in 87.07% F1 score, and object detection with Faster R-CNN resulted in 41.18% F1 score [28]. 

The literature reveals that most of the researchers have applied CNN in the retrospective study to detect and classify oral cancer. It can be observed that most of the classification tasks were binary classification, where the features from the image like colour, shape, and texture are used. Even though, CNN is a powerful visual model for image recognition tasks, some researchers used deep CNNs which outperform conventional CNN in classification tasks.

Chan et al. proposed an innovative deep CNN combined with a texture map for oral cancer detection. The proposed model consists of two collaborative branches, one to perform oral cancer detection, and a second one to perform region of interest (ROI) marking and semantic segmentation. The experimental results of detection, based on a wavelet transform, are up to 96.87% for sensitivity and 71.29% for specificity [29]. Fraz et al. demonstrated a network for simultaneous segmentation of microvessels and nerves of oral cancer. The dataset used consisted of 7780 H&E-stained histology images, size 512 × 512 pixels, extracted from 20 WSIs of oral carcinoma tissue. The proposed block-based pyramid pooling deep neural network outperforms other deep CNN for semantic segmentation [30]. 

Most of the segmentation tasks were performed using deep CNN architectures on histopathological images. A shortcoming of the prementioned studies is that they were trained to determine a binary state of carcinoma or non-carcinoma. Based on a literature study only Das et al. proposed a deep learning model for the classification of cells into multiple classes in epithelial tissue of OSCC. The dataset consisted of image patches derived from whole slide biopsy images. Proposed CNN model resulted in 97.5% accuracy [31]. 

According to the presented studies different AI approaches have proven successful for clinical analysis [21,22,23,24,25,26,27,28,29]. However, in the terms of histological analysis of OC, fewer studies have been conducted since histopathological examination is highly invasive [30,31]. Exhaustive literature study reveals that, at the time when this research was performed, no work has been done on multiclass grading along with segmenting of OSCC using whole-slide histopathology images obtained by biopsy and stained with marker protein.

## 2. Materials and Methods

**Figure 1:**
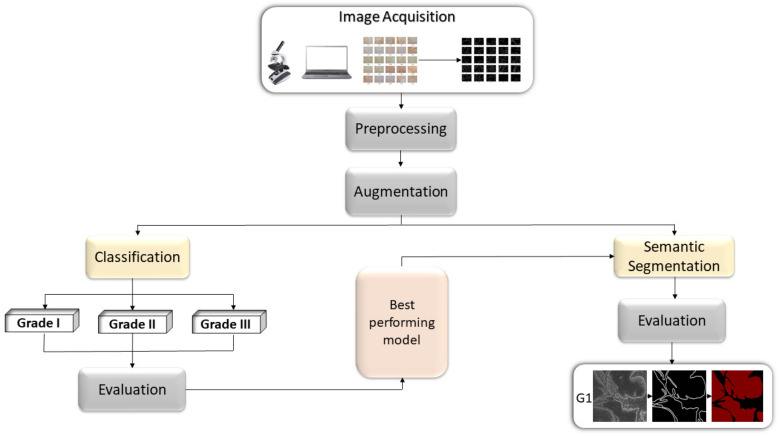
Block diagram representation of the proposed methodology.

The overall workflow of the research can be described as follow; first, the original data will be augmented and used as an input variable in order to perform multiclass classification by utilizing AI-based models. After evaluating the performance of the models, the obtained results will be compared, and the best performing model will be selected. Second, the input data will be decomposed using stationary wavelet transform in order to obtain wavelet coefficients. The obtained coefficients will be preprocessed and the data will be reconstructed. Afterwards, the data preprocessed with SWT will be used to train the selected model, and the obtained results will be compared. Third, the selected model will be used as Deeplabv3+ backbone, by which, semantic segmentation into two classes will be performed. Fourth, the impact of data preprocessing on the model performance and robustness will be examined. Lastly, the best performing configuration in terms of semantic segmentation will be used to show predictions of the epithelial and stromal tissue on three samples from new, unseen data. The overview of the proposed framework is given in Figure 1.

### 2.1. Dataset Description

For this research, 322 histology images with 768 × 768-pixel size have been used to create a dataset. The formalin-fixed, paraffin-embedded oral mucosa tissue blocks of histopathologically reported cases of OSCC were retrieved from the archives of the Clinical Department of Pathology and Cytology, Clinical Hospital Center in Rijeka. Sample slides were reviewed by two unbiased pathologists and classified following the 4th edition of the World Health Organization (WHO) classification of Head and Neck tumors [32] and 8th edition of the AJCC Cancer Staging Manual [33].

The paraffin blocks tissues were selected from the patients with primary invasive squamous cell carcinomas, where the invasion of the epithelium of the oral cavity through the basement membrane was confirmed with certainty. All patients who received preoperative chemo- or radiation-therapy, as well as patients with in situ carcinoma and relapsed or second primary OSCC were excluded from the research.

**Figure 2:**
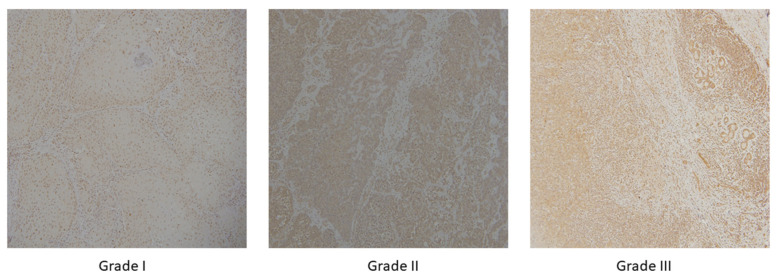
OSCC group of well-differentiated OSCC (**grade I**), moderately differentiated OSCC (**grade II**) and poorly differentiated OSCC (**grade III**) with magnification × 10.

Kappa coefficient was used to determine the degree of agreement between the pathologists. The value of Kappa coefficient was found to be 0.94. In accordance with the aforementioned classification, images have been divided into three classes: well-differentiated (grade I), moderately differentiated (grade II), and poorly differentiated (grade III) OSCC, as shown in Figure 2. Additionally, the segmentation masks were prepared by a health professional and validated by another independent pathologist.

Briefly, 4 μm sized paraffin-embedded tissue sections were deparaffinized in tissue clear solution, rehydrated in a series of different concentrations of alcohol and stained using monoclonal mouse anti-MT I + II antibody ((clone E); DAKO, Santa Clara, CA, USA; diluted 1:100 in PBS with 1% BSA)) and polyclonal rabbit anti-megalin (Santa Cruz Biotechnology, Dallas, TX, USA; diluted 1:100 in PBS with 1% BSA) using a standard protocol [34]. Immunoreaction was visualized by adding peroxidase substrate solution containing diaminobenzidine (DAB). Slides were afterwards stained with hematoxylin (Sigma-Aldrich, Munich, Germany), dehydrated and mounted with Entellan (Sigma-Aldrich).

Images were captured using the light microscope (Olympus BX51, Olympus, Tokyo, Japan) equipped with a digital camera (DP50, Olympus) and transmitted to a computer by CellF software (Olympus). Furthermore, images were captured at 10 × objective lenses. 

Corresponding clinic-pathological reports of the patients were collected and used for pTMN classification [33]. Patient demographic information included age at the time of diagnosis, sex, smoking. Patients were adults, where the median age was 64, and 69% of them were smokers. As seen in Table 1. more patients were male (65%) while 35% were female. The least number of patients were diagnosed with grade III (17%) whereas 50% were diagnosed with grade I. In more patients (54%) presence of metastases in the lymph nodes were excluded.

Deep CNNs are heavily reliant on a large number of samples in order to achieve satisfactory performance and avoid overfitting. Since domains, such as medical image analysis, do not have access to a large number of samples very often, it is necessary to perform augmentation techniques, by which, the size and quality of the data can be significantly increased [35]. Due to the aforementioned neural network demand and limited availability of the data, augmentation techniques are performed to artificially increase the number of samples. 

Geometrical transformations used for the augmentation procedure are: 90 degrees anticlockwise rotation, 180 degrees anticlockwise rotation, 270 degrees anticlockwise rotation, horizontal flip, horizontal flip combined with 90 degrees anticlockwise rotation, vertical flip, and vertical flip combined with 90 degrees anticlockwise rotation. Since newly generated data are variations of the original data, the augmentation procedure is utilized only for the creation of the training samples, thereby, testing samples are not augmented. 

Due to the high-imbalance of OSCC classes, stratified 5-fold cross-validation is used to estimate the performance of AI-based models. This way, the representation of each class across each test fold is approximately the same [36]. Accordingly, within each fold, the first part (approximately 80% of each class) is augmented and used for model training, while the second part (approximately 20% of each class) is used to evaluate the performance of the trained models.

**Figure 3:**
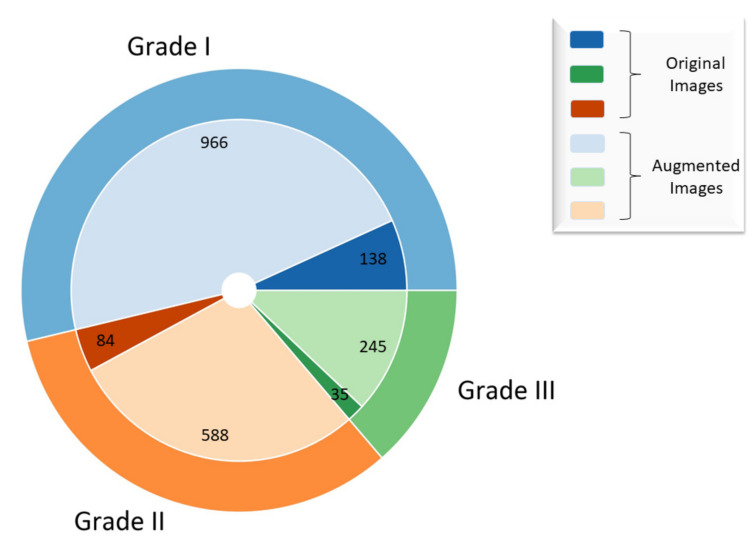
Representation of original and augmented dataset.

By utilizing the aforementioned transformations, a new training set with additional 1799 images has been created, which gives a total of 2056 images as shown in Figure 3.

### 2.2. Preprocessing Method Based on Stationary Wavelet Transform and Mapping Function

The wavelet transform (WT) is a powerful technique commonly used in data preprocessing [37]. Applying WT, data can be represented at different scales and frequencies. Furthermore, it is a useful tool for describing the image in multiple resolutions. Wavelet transform of signal x(t) can be defined as [38]:

(1) $$X\left(\tau ,a\right)=\frac{1}{\sqrt{\left|a\right|}}\overset{\infty }{\underset{-\infty }{\int }}x\left(t\right)\psi^{*}\left(\frac{t-\tau }{a}\right)\mathrm{dt},$$

where a is the dilation, ψ is analysing wavelet, and τ is translation parameter. By performing WT, low-frequency components (i.e., approximation coefficients) and high-frequency components (i.e., detail coefficients) can be obtained [39]. The discrete wavelet transform (DWT) of signal x[m] can be calculated as follows [38]:

(2) $$X\left[k,l\right]=2^{-\frac{k}{l}}\overset{\infty }{\underset{m=-\infty }{\sum }}x\left[m\right]\psi \left[2^{-k}m-l\right].$$

When dealing with images, DWT is applied in each dimension separately; therefore, the image is divided into four subbands LL, LH, HL, and HH where LL represents approximation coefficients while LH, HL, and HH represent detail coefficients of an image [40]. DWT reduces the computation time and can be implemented easily, but it also suffers in terms of shift-invariance and decimation. In order to overcome aforementioned drawbacks, the research utilises stationary wavelet transform (SWT) by which histopathology images can be decomposed. 

The advantages of SWT are as follows [41]:

- no decimation step—provides redundant information,
- better time-frequency localization, and
- translation-invariance.

**Figure 4:**
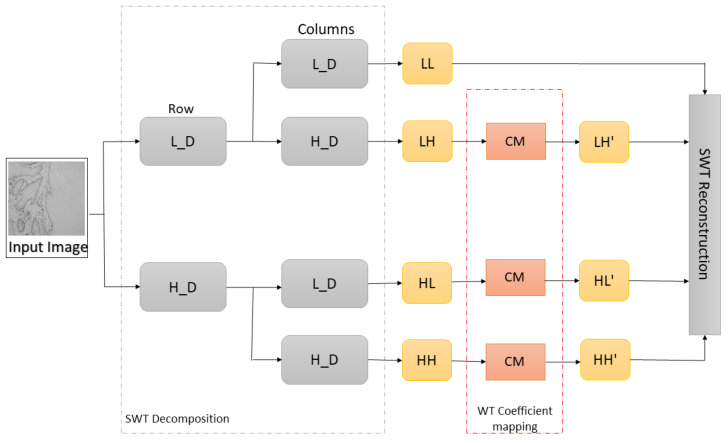
Representation of SWT decomposition, wavelet coefficient mapping, and SWT reconstruction (L_D—low pass filter, H_D—high pass filter, LL—approximation coefficients, LH—horizontal coefficients, HL—vertical coefficients, HH—diagonal coefficients, and CM–coefficient mapping function).

After the SWT decomposition process, obtained coefficients are weighted using a mapping function. This way important features of an image can be further enhanced. The following considerations are taken into account when determining the mapping function; First–coefficient mapping is performed only on detail coefficients. Second–details with high, as well as details with low coefficient values preserve valuable information, thereby, they are heavily weighted. Wavelet coefficient mapping function can be mathematically defined as follows:

(3) $$y_{i,\;j}={\mathrm{aw}}_{i,\;j}^{3}+{\mathrm{bw}}_{i,\;j}^{2}+{\mathrm{cw}}_{i,\;j}+d,$$

where a, b, c, and d represent constants, $w_{i,\;j}$ is an input coefficient, while $y_{i,\;j}$ is a coefficient after mapping. After the coefficient mapping process, the SWT reconstruction is performed with approximation and weighted SWT coefficients in order to obtain an enhanced image. The process of SWT decomposition–coefficient mapping–SWT reconstruction is shown in Figure 4.

The quality of weighted coefficients directly depends on mapping function constants and wavelet function; therefore, a careful selection of these parameters is necessary. Additionally, the selection of the parameters also depends on the type of input data and expected output. More traditional approaches for parameter determination such as random search or grid search can sometimes be infeasible since evaluating each point in large search-spaces is extremely costly (computationally) [42]. These methods do not consider evaluated performances of past iterations when selecting the next configuration of parameters, often resulting in spending time on evaluating function with a poor selection of parameters. In contrast, the Bayesian approach for determining parameters of mapping function selects the next configuration of parameters based on results obtained in past iterations [43]. This way, convergence to the best solution can be achieved in fewer iterations, thus outperforming more traditional methods.

In order to determine optimal values of wavelet coefficient mapping function constants $\left(a,\;b,\;c,\;d\right)$ and wavelet function, Bayesian optimization has been used. The domain of mapping function constants over which to search is defined and shown in Table 2.

### 2.3. AI-Based Models

In this subsection, a brief overview of deep CNN architectures suitable for OSCC classification problem is given.

#### 2.3.1. Xception

**Figure 5:**
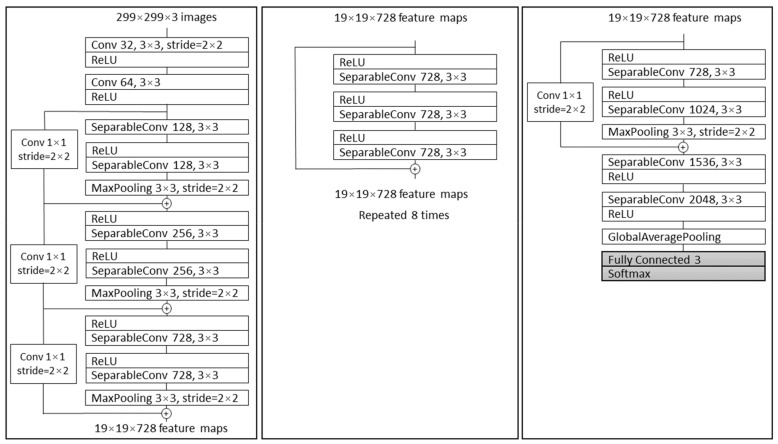
The Xception architecture; first, the data propagate through entry flow (first box), then through middle flow (second box) and repeats eight times. In the end, data propagate through the third box which represents exit flow [44].

Chollet demonstrated a novel architecture named Xception, based on Inception V3. Compared to Inception V3 on ImageNet, Xception resulted in larger performance improvement [44]. In a conventional CNN, convolutional layers search through depth and space for correlation. Xception takes few steps further with mapping the spatial correlations for each output channel separately and performing 1 × 1 depth-wise convolution to capture cross-channel correlation. Xception architecture consists of 36 convolutional layers which are structured in 14 modules [44]. All the modules have linear residual connections around them, not including the first and last module, as shown in Figure 5.

#### 2.3.2. ResNet50 and −101

As neural networks become deeper, they become difficult to train due to the notorious vanishing gradient problem. In order to ease the training of deep neural networks, He et al. propose a residual network (ResNets) [45]. They refined the residual block along with the pre-activation variant of the residual block where through the shortcut connections vanishing gradients can flow unimpededly to any other previous layer. In ResNet50 architecture, every 2-layer block is replaced in the 34-layer network with a 3-layer bottleneck block, which results in 50 layers, while ResNet101 architecture is constructed using more 3-layer blocks as shown in Table 3. The number of parameters for ResNet50 and ResNet101 totals 23,888,771 and 42,959,235, respectively. On the ImageNet dataset, He et al. proved that ResNets with achieved 3.57% error outperforms other architectures on the ILSVRC classification task [45].

#### 2.3.3. MobileNetv2

Sandler et al. introduced in their article [46] the mobile architecture called MobileNetv2 which enhances the performance of mobile models. It builds on MobileNetv1’s ideas by utilizing depthwise separable convolution as efficient building blocks. Compared to conventional residual models that use an extended input representation, MobileNetv2 as input to the residual block use thin bottleneck layers. The MobileNetV2 architecture includes the initial fully convolution layer with 32 filters and 19 residual bottleneck layers. The detailed architecture structure is shown in Table 4.

MobileNetv2 for classification task was compared with MobileNetv1, NASNet-A, and ShuffleNet on the ImageNet dataset. The results show that presented architecture with specific design variations improves the state-of-the-art for a wide range of performance points [45].

### 2.4. DeepLabv3+

**Figure 6:**
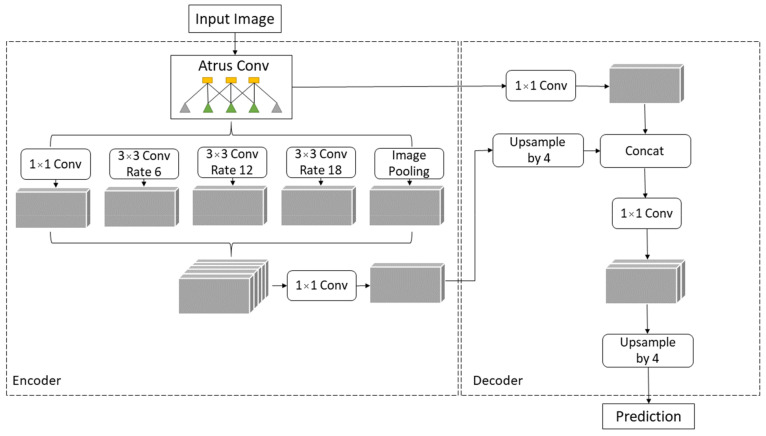
DeepLabv3+ Encoder-Decoder architecture.

The process of assigning a semantic label to each part of the image is called semantic segmentation. Chen et al. proposed the DeepLab system as state-of-the-art at PASCAL VOC 2012 semantic segmentation task and based on the experimental results they achieved 79.7% mIOU on the test set [47]. Later, Chen et al. presented the DeepLabv3 system that improves their previous versions of DeepLab. Without DenseCRF postprocessing, the presented system attains a performance of 85.7% mIOU on the PASCAL VOC 2012 dataset [48]. DeepLabv3+ is an extended version of DeepLabv3 that includes a decoder module to refine the result of segmentation. Due to Chen et al.’s paper, DeepLabv3+ without any postprocessing achieves a performance of 89% mIOU on the PASCAL VOC 2012 dataset [49]. DeepLab and DeepLabv3 use Atrous Spatial Pyramid Pooling (ASPP) to encode multi-scale contextual information while DeepLabv3+ combines encoder-decoder pathway and ASPP, as shown in Figure 6, intending to achieve more precise delineation of object boundaries [50].

The architectures described in Section 2.3 (Xception, ResNet50, ResNet101, MobileNetv3) can be used as DeepLabv3+ backbones.

### 2.5. Evaluation Criteria

In order to evaluate and analyse the obtained results, it is necessary to describe evaluation metrics. Statistical measures such as micro- and macro- area under the curve (AUC) are adopted to evaluate the classification performance of models. In the second stage, semantic segmentation performances are evaluated using mean intersection-over-union (mIOU), Dice coefficient (F1), accuracy (ACC), precision, sensitivity, and specificity.

The AUC is an evaluation metric for calculating the performance of the binary classifier. To extend AUC to multiclass classification, it is necessary to conceptualize the problem as a binary classification problem, by using One vs. All technique, which means that one class is classified against all other classes. Micro averaging of true positive rate (TPR) calculates the number of correct classifications for each class and uses it as the numerator, while for the denominator it uses the total number of samples. Furthermore, fallout or false positive rate (FPR) calculates the ratio of incorrect classifications for each class and the total number of samples [51]. The mathematical representation of micro averaging is defined as follows

(4) $${\mathrm{TPR}}_{\mathrm{micro}\;}=\;\frac{\sum_{i=1}^{k}{\mathrm{TP}}_{i}}{\sum_{i=1}^{k}{\mathrm{TP}}_{i}+\sum_{i=1}^{k}{\mathrm{FN}}_{i}}$$

and:

(5) $${\mathrm{FPR}}_{\mathrm{micro}\;}=\frac{\sum_{i=1}^{k}{\mathrm{FP}}_{i}}{\sum_{i=1}^{k}{\mathrm{FP}}_{i}+\sum_{i=1}^{k}{\mathrm{TN}}_{i}}\;,\;$$

by which, AUC_micro_ can be calculated. TP represents true positives, i.e., cases where the predicted and actual values are positive. TN represents true negatives, cases where the actual and predicted values are negative. False negatives (FN) capture cases when the prediction is negative and the actual value is positive. Furthermore, FP represents false positives, where the prediction is positive, and the actual value is negative [52].

Macro averaging for k classes compute the metrics individually for each class and averages results together. AUC_macro_ is based on the calculation of TPR_macro_ as well as FPR_macro_ and can be calculated as follows

(6) $${\mathrm{TPR}}_{\mathrm{macro}\;}=\;\frac{\sum_{i=1}^{k}{\mathrm{TPR}}_{i}}{k}$$

and:

(7) $${\mathrm{FPR}}_{\mathrm{macro}\;}=\;\frac{\sum_{i=1}^{k}{\mathrm{FPR}}_{i}}{k}.$$

Higher values of AUC_macro_ and _-micro_ measure will result in better classification performance of the model. 

One of the most used metrics for semantic segmentation is IOU, also known as the Jaccard Index. If the previously introduced terms TP, TN, FP, and FN are used, the Jaccard index can be defined as [53]:

(8) $$\mathrm{IOU}=\;\frac{\mathrm{TP}}{\mathrm{TP}+\mathrm{FP}+\mathrm{FN}}.$$

Since this research deals with multiple classes (more than one), mean IOU (mIOU) is used as a metric. It can be calculated as a ratio between a total number of IOU-s for each semantic class and total number of classes. Dice coefficient is positively correlated with the IOU. It is an overall measure of a model’s accuracy and can be defined as [54]:

(9) $$F1=\;\frac{2\mathrm{TP}}{2\mathrm{TP}+\mathrm{FP}+\mathrm{FN}}.$$

F1 score ranges in 0–1 where the model with low false positives and low false negatives performs well. Accuracy measure points out what percentage of the pixels in the image are assigned to the correct class, and can be calculated as follows [55]:

(10) $$\mathrm{ACC}=\;\frac{\mathrm{TP}+\mathrm{TN}}{\mathrm{TN}+\mathrm{TP}+\mathrm{FN}+\mathrm{FP}},$$

while precision shows the percentage of the results which are relevant and can be expressed as [54]:

(11) $$\mathrm{Precision}=\;\frac{\mathrm{TP}}{\mathrm{TP}+\mathrm{FP}}.$$

Mathematically, sensitivity and specificity can be calculated as the following [56]

(12) $$\mathrm{Sensitivity}=\;\frac{\mathrm{TP}}{\mathrm{TP}+\mathrm{FN}}$$

and:

(13) $$\mathrm{Specificity}=\;\frac{\mathrm{TN}}{\mathrm{TN}+\mathrm{FP}}.$$

Higher values of performance measures defined by Equations (8)–(13) mean better segmentation performance of the model.

## 3. Results

**Figure 7:**
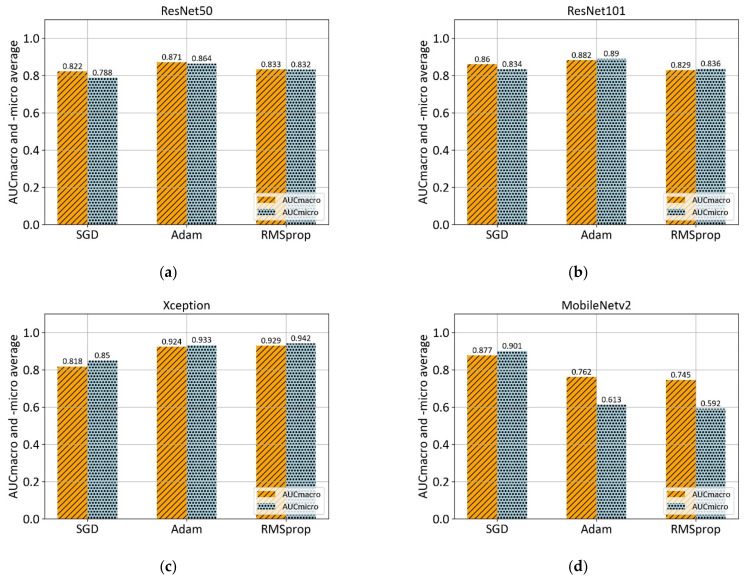
Comparison of mean AUC_macro_ and _-micro_ values of three different optimizers (SGD, ADAM, and RMSprop) on pre-trained models: (**a**) ResNet50; (**b**) ResNet101; (**c**) Xception; and (**d**) MobileNetv2.

This section demonstrates the experimental results obtained at each step of the proposed methodology. The first experimental results are achieved with Xception, ResNet50, ResNet101, and MobileNetv2 architectures which are pre-trained on ImageNet. Each model architecture is trained with three optimizers: stochastic gradient descent (SGD), Adam, and RMSprop, as shown in Figure 7.

According to the 5-fold cross-validation results, the Adam optimizer achieves the highest AUC_macro_ and _-micro_ values in the case of ResNet50, ResNet101, and MobileNetv2 architectures. However, RMSprop optimizer in a combination with Xception architecture achieves the overall highest values of AUC_macro_, and _-micro_. Summarized mean values of performance measure along with corresponding standard deviation for each model architecture is shown in Table 5.

The best results obtained in the first step were achieved when two additional layers were added at the end of the base Xception architecture. After the separable convolutional layer, and ReLU activation function, the global average pooling layer followed by the output layer with three neurons and Softmax activation function were added. This way, a classification of OSCC into three classes was enabled. The training process was divided into two stages; the first stage where only added layers were trainable while the others were frozen, and the second stage, where all layers were trainable except the last two added layers. In the first stage, the training process was performed with a learning rate of 0.001 and learning rate decay of 1 × 10^−6^. The training process in the second stage was performed with a learning rate of 0.0001 and the same learning rate decay of 1 × 10^−6^.

**Figure 8:**
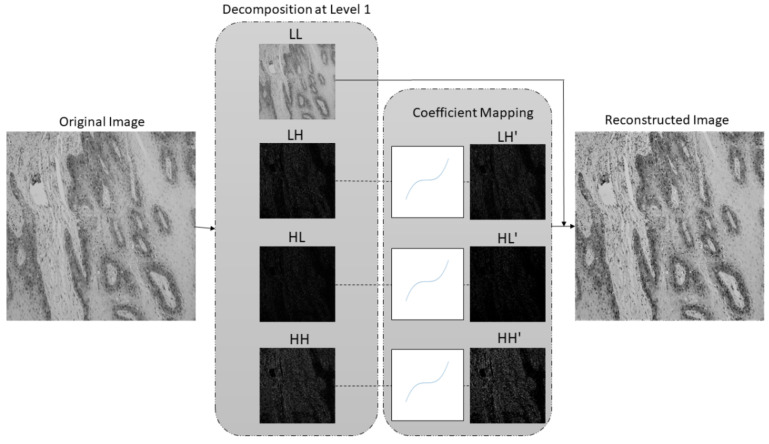
SWT decomposition at level 1 using Haar wavelet along with coefficient mapping, and SWT reconstruction.

The second step of the proposed methodology is data preprocessing using Stationary Wavelet Transform, where original histopathology images were decomposed at level 1 using Haar, sym2, db2, and bior1.3 wavelet functions. After the decomposition process, high-frequency wavelet coefficients LH, HL, HH are weighted using mapping function defined by Equation (3), which resulted in new, modified LH’, HL’, HH’ subbands. Modified subbands along with unmodified LL subband were used for SWT reconstruction in order to obtain input image for AI model, as shown in Figure 8.

By utilizing Bayesian optimization, the goal was to find optimal values of wavelet mapping function constants, by which, the maximal value of performance measure is achieved. In the case of this research, AUC_micro_ performance measure was monitored during the process of optimizing. Each Bayesian iteration involved data preprocessing with a defined set of mapping function constants, model training process, and performance evaluation. After 25 steps of random exploration and 20 steps of Bayesian optimization, the best performing constant configuration was obtained as shown in Table 6.

**Figure 9:**
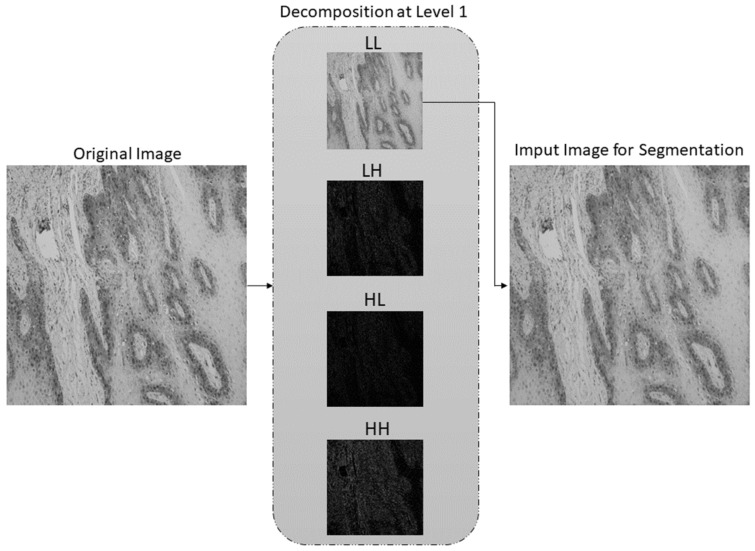
SWT decomposition at level 1 using Haar wavelet function. LL subband is used as an input image for semantic segmentation.

After multiclass grading of OSCC from histopathological images, the third step is semantic segmentation of the epithelial and stromal tissue. In contrast to the aforementioned preprocessing approach for multiclass classification, the data preprocessing in the case of semantic segmentation utilizes only a low-frequency subband. Therefore, after one-level decomposition utilizing the SWT with Haar, sym2, db2, and bior1.3 wavelet functions, only low-frequency coefficients with applied Greys colormap were used as input for the AI model. SWT decomposition of an image at level one using Haar wavelet is shown in Figure 9.

According to the results presented in Table 5, Xception_65 was used as DeepLabv3+ backbone in order to perform semantic segmentation. The model was pre-trained on the Cityscapes dataset before the training on the oral carcinoma dataset was performed. As model input, original images and SWT approximations were used along with corresponding ground truth masks. In the training process, ASPP in a configuration with atrous rates of 12, 24, 36 was used while the output stride was set to 8. Additionally, decoder output stride was set to 4. Performances of the models achieved on 5-fold cross-validation are shown in Table 7.

**Figure 10:**
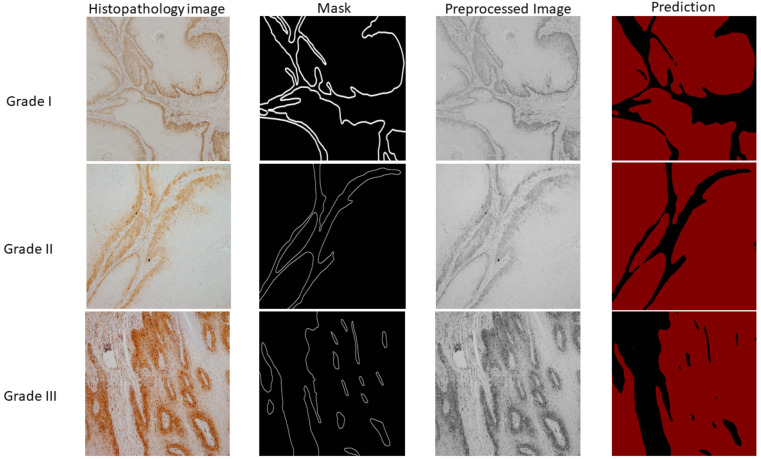
Visual representation of histopathology images, ground truth masks, preprocessed images, and semantic segmentation results. The first column represents samples of OSCC obtained by the clinician while the second column is corresponding ground truth mask. The third column represents samples after preprocessing which are afterwards used as input variables for semantic segmentation. Finally, the last column shows the prediction for three cases (Grade I, II, and III) where the black colour represents stromal tissue and the red colour represents epithelial tissue.

By using data preprocessed with SWT at level one using a Haar wavelet function and a fully-trained DeepLabv3+ model, the predictions were made for the three cases of new and unseen data, as visually represented in Figure 10. Each sample from visual representation corresponds to a specific grade of OSCC.

AI experiments were performed using Python on a GPU-based High Performance Computing (HPC) server. The server consists of two Intel Xeon Gold CPUs (24 C/48 T, at 2.4 GHz), 768 GB of ECC DDR4 RAM, and five Nvidia Quadro RTX 6000 GPUs, with 24 GB of RAM, 4608 CUDA and 576 Tensor cores.

## 4. Discussion

The diagnosis of OSCC is based on a histopathological assessment of altered oral mucosa which despite high subjectivity, still is the most reliable method of diagnosing oral carcinoma. The pathologist evaluates and grades the tumor depending on resemblance to the normal oral squamous epithelium as well, moderately or poorly differentiated [57]. Histopathological characteristics of well differentiated carcinoma are tumor islands with a central substantial amount of keratinization, keratin pearls. Moderately and poorly differentiated squamous cell carcinoma show more prominent nuclear and cellular pleomorphism, and prominent mitotic figures that can be abnormal, respectively. Usually, it is easy to identify an oral carcinoma by the invasion of the epithelial cell through the basement membrane border into connective tissues [57]. However, histopathological classification of oral carcinoma can be challenging due to heterogeneous structure and textures, and the presence of any inflammatory tissue reaction. With the help of artificial intelligence-aided tools, the automatic classification of histopathological images can not only improve objective diagnostic results for the clinician but also provide detailed texture analysis in order to get an accurate diagnosis [58]. 

This research proposes an AI-based system for multiclass grading and semantic segmentation of OSCC. The proposed system is compared with similar approaches presented in the literature [31,59] to validate feasibility. 

The training process, in the case of multiclass grading, was performed in two stages as described in results section. The purpose of the two-stage learning process is to achieve better performance and at the same time increase the robustness of trained models. Since two additional layers were added at the end of the original Xception architecture, their weights were randomly initialized; therefore, training with a learning rate value of 0.001 was performed. However, in the second stage of the training process, the learning rate was set to a lower value of 0.0001. This way, the weights of layers pre-trained on ImageNet were only adapted to the new problem of multiclass grading.

From the presented results, in the case of multiclass grading with no preprocessing, it can be concluded that high values of 0.929 AUC_macro_ and 0.942 AUC_micro_ are achieved with a combination of Xception architecture and RMSprop optimizer. Furthermore, ResNet50 in a combination with the Adam optimizer showed AUC_macro_ and _-micro_ values of 0.871 and 0.864, respectively, which is slightly lower than ResNet101 performance (0.882 AUC_macro_ and 0.890 AUC_micro_). However, ResNet101—Adam was worst-performing in terms of standard deviation with values of ±0.125, and ±0.112. Lowest values of standard deviation were obtained in the case of MobileNetv2 architecture in a combination with Adam optimizer.

Since important features for distinguishing the differences between OSCC gradings are mostly contained in high-frequency components of the image, the wavelet coefficient mapping function was proposed. With the help of SWT, the image was decomposed on the LL, LH, HL, and HH subbands which allowed coefficient weighting. Mean values of the high-frequency components were located around zero; therefore, constants of coefficient mapping function were determined in a way that features with high- and low-coefficient values were enhanced. SWT reconstruction process with LL, LH’, HL’, and HH’ subbands, resulted in images with enhanced high-frequency features.

If the performances, in the case of multiclass grading with preprocessing, are compared, it can be seen that all of the presented configurations achieved AUC_macro_ value of 0.947 and AUC_micro_ value of 0.954 or higher. Moreover, when all results are summed up, it can be noticed that the highest values of performance measure are achieved using the proposed methodology with coefficient mapping function constants a, b, c, and d with values of 0.091, 0.0301, 0.0086, and 0.3444, respectively, and db2 as wavelet function. Performance of the proposed model in terms of AUC_macro_ and AUC_micro_ values is 0.963 ± 0.042 and 0.966 ± 0.027, respectively; therefore, it can be concluded that not only performance measure was increased, but also the values of standard deviation were decreased. A decrease in standard deviation value resulted in increased robustness of the model.

The foremost task for OSCC diagnosis and treatment plan is accurate histopathology examination of samples to detect morphological changes of the tumor cells. Histopathological grading is focused on the tumor cells features like nuclear pleomorphism, mitotic activity, depth of invasion, tumor thickness and degree of differentiation [60]. However, recent research showed the importance of the microenvironment in tumor progression and poor prognosis. The stroma-rich tumors showed association with unfavourable prognosis compered to stroma poor tumors, due to this the tumor-stroma ratio (TSR) could be useful prognostic outcomes factor [61]. Heikkinen et al. suggested that the degree of stromal tumor-infiltrating lymphocytes (TILs) can be used like prognostic features, while other studies based on immunohistochemistry emphasize the prognostic value of different subtypes of immune cells and lymphocytes [62,63,64]. 

The conventional practice, histopathological assessment on light microscopy is time-consuming, tedious, and relies on the experience of pathologists. The characteristic of the microscope (resolution, light source, and the lens) and preparation of the tissue samples may hamper diagnosis and affect manual judgment. The histological parameters can be investigated under different magnification (4×, 10×, 20×, and 40×). Literature reveals that most of the segmentation research has been performed on 40× magnified images [65,66,67,68]. The 40× magnification allows the pathologist to visual features of the nucleus of cells and cell structure in the tissue as opposed to 10× magnification but provides more incomprehensive picture of the tumors.

Recently, many computer-aided tools for medical image analysis show significant progress towards better healthcare services. The second stage of the proposed AI-based system is semantic segmentation which is a mandatory step towards tumor microenvironment analysis. The semantic segmentation of epithelial and stromal tissue is performed on 10× magnification histopathology images. 

In contrast to the preprocessing in the case of multiclass grading, preprocessing for semantic segmentation implies using only low-frequency coefficients, i.e., LL subband, while the high-frequency subbands are discarded. Therefore, by using extracted low-frequency features, DeepLabv3+ in a combination with Xception_65 as backbone achieved satisfactory results in the case of multiclass semantic segmentation of tumor tissue. From the obtained results it can be noticed that the segmentation model in a combination with low-frequency features outperformed the model which used original, non-preprocessed data as input. In terms of performance measures, the highest mIOU (0.879), F1 (0.955), and Accuracy (0.941) values are achieved using low-frequency features obtained at SWT decomposition level-one using Haar wavelet. However, in terms of Precision and Specificity performance measures, data preprocessed with db2 wavelet function outperforms others with values of 0.952 (Precision), and 0.911 (Specificity). When Sensitivity measure is observed, non-preprocessed data achieved the highest value of 0.967.

Presented results indicate that segmenting of epithelial and stromal tissue has a great potential in the quantification of qualitative clinic-pathological features in order to predict tumor aggressiveness and metastasis. Segmented areas will be used in future work for analysing the tumor microenvironment, where the stroma is essential for the maintenance of epithelial tissue. Stromal and epithelial regions of the OSCC have a different impact on the disease progression which is important for the histopathology image analysis.

AI computer-aided systems for analysing the tumor microenvironment might contribute to modified treatment planning, improving prognosis and survival rates, and maintaining a high-quality life of patients.

## 5. Conclusions

Histology grading is a way to classify cancer cells based on tissue abnormality. It relies on the subjective component of the clinician and as such may adversely affect appropriate treatment methods and outcomes of the patient. This research highlights the huge potential of the application of image processing techniques with the aid of Artificial Intelligence algorithms in order to achieve an effective prognosis of OSCC and increase the chance of survival among people.

In the first stage of the research, the authors demonstrate the integration of deep convolutional neural networks with stationary wavelet transform along with wavelet coefficient mapping function for multiclass grading of OSCC. From obtained results, it can be concluded that integration of Xception and SWT resulted in the highest classification values of 0.963 AUC_macro_ and 0.966 AUC_micro_ with the lowest standard deviation of ±0.042 and ± 0.027, respectively. 

In the second stage, semantic segmentation was performed. Xception_65 as DeepLabv3+ backbone, integrated with low–frequency subband resulted in 0.879 ± 0.027 mIOU, 0.955 ± 0.014 F1 score and 0.941 ± 0.015 accuracy. Segmentation of the tumor on the epithelial and stromal regions is the initial step in the study of the tumor microenvironment and its impact on the disease progression. 

Based on the results of the proposed methodology, a two-stage AI-based system has been proven successful in terms of multiclass grading as well as segmenting of epithelial and stromal tissue and has a great potential in the clinical application in prediction of tumor invasion and outcomes of a patient with OSCC. Since the limitation of this research was data availability, future work should use a dataset with more histopathology images, to achieve a more robust system. The presented approach will be the first step in analysing the tumor microenvironment, i.e., tumor-stroma ratio and segmentation of the microenvironment cells. The main idea is to develop an advanced automatic prognostic system that could analyse parameters such as cell shape and size, pathological mitoses, tumor-stroma ratio and distinction between early- and advanced-stage OSCCs. Moreover, try to create predictive algorithms that would improve prognostic indicators in clinical practice and thus improve therapeutic treatment for the patient.

## Figures and Tables

**Table 1:** Characteristic of the patients include sex, age, smoking habits, presence of metastases in the lymph nodes, and histological grade of carcinoma.

Characteristic of the Patients		%
Sex	F	35
	M	65
Age	To 49	6
	50–59	13
	60–69	58
	+70	23
Smoking	Y	69
	N	31
Lymph Node Metastases	Y	46
	N	54
Histological Grade (G)	I	50
	II	33
	III	17

**Table 2:** List of hyperparameters used in the Bayesian optimization process.

Hyperparameter	Possible Parameters
a	0–0.1
b	0–0.1
c	0–0.1
d	0.001–1
Wavelet function	Haar, sym2, db2, bior1.3

**Table 3:** ResNet50 and ResNet101 architecture representation.

Layer	Output	Layers	ResNet50	ResNet101
			Number of Repeating Layers	
Conv1	112 × 112	7 × 7, 64, stride 2	×1	×1
		3 × 3 max pool, stride 2	×1	×1
Conv2_x	56 × 56	1 × 1, 64	×3	×3
		3 × 3, 64		
		1 × 1, 256		
Conv3_x	28 × 28	1 × 1, 128	×4	×4
		3 × 3, 128		
		1 × 1, 512		
Conv4_x	14 × 14	1 × 1, 256	×6	×23
		3 × 3, 256		
		1 × 1, 1024		
Conv5_x	7 × 7	1 × 1, 512	×3	×3
		3 × 3, 512		
		1 × 1, 2048		
	1 × 1	Flatten	×1	×1
		3-d Fully Connected		
		Softmax		

**Table 4:** MobileNetv2 architecture; each row represents a sequence of at least 1 identical layer, repeated n times. The number c of output channels is the same for each layer in the same sequence. The first layer of each sequence consists of a stride s while all the rest use stride 1. The expansion factor t is used for the input size.

Input	Operator	Expansion Factor (t)	Number of Output Channels (c)	Repeating Number (n)	Stride (s)
224 × 224 × 3	conv2d	-	32	1	2
112 × 112 × 32	bottleneck	1	16	1	1
112 × 112 × 16	bottleneck	6	24	2	2
56 × 56 × 24	bottleneck	6	32	3	2
28 × 28 × 32	bottleneck	6	64	4	2
14 × 14 × 64	bottleneck	6	96	3	1
14 × 14 × 96	bottleneck	6	160	3	2
7 × 7 × 160	bottleneck	6	320	1	1
7 × 7 × 320	conv2d 1 × 1	-	1280	1	1
7 × 7 × 1280	avgpool 7 × 7	-	-	1	-
1 × 1 × 1280	fully connected (Softmax)	-	3	-	

**Table 5:** Performance of different algorithms using AUC_macro_ and _-micro_ as evaluation metrics along with standard deviation (σ).

Algorithm	AUC_macro_ ± σ	AUC_micro_ ± σ
ResNet50	0.871 ± 0.105	0.864 ± 0.090
ResNet101	0.882 ± 0.125	0.890 ± 0.112
Xception	0.929 ± 0.087	0.942 ± 0.074
MobileNetv2	0.877 ± 0.062	0.900 ± 0.049

**Table 6:** Constants of coefficient mapping function obtained using Bayesian optimization along with corresponding 5-fold cross-validation performance.

Parameters					Xception + SWT	
a	b	c	d	Wavelet	AUC_macro_ ± σ	AUC_micro_ ± σ
0.0084	0.0713	0.0599	0.0566	sym2	0.956 ± 0.054	0.964 ± 0.040
0.0091	0.0301	0.0086	0.3444	db2	0.963 ± 0.042	0.966 ± 0.027
0.0063	0.0021	0.0771	0.3007	db2	0.947 ± 0.092	0.954 ± 0.069
0.0081	0.0933	0.0469	0.2520	haar	0.952 ± 0.056	0.958 ± 0.050
0.0053	0.0575	0.0649	0.1694	bior1.3	0.962 ± 0.050	0.965 ± 0.046

**Table 7:** Performance of DeepLabv3+ with Xception_65 as backbone trained with data preprocessed with different wavelet functions.

		mIOU ± σ	F1 ± σ	Accuracy ± σ	Precision ± σ	Sensitivity ± σ	Specificity ± σ
DeepLabv3+ & Xception_65	Original	0.864 ± 0.020	0.933 ± 0.058	0.934 ± 0.012	0.933 ± 0.019	0.967 ± 0.013	0.873 ± 0.017
	sym2	0.874 ± 0.037	0.953 ± 0.016	0.939 ± 0.019	0.950 ± 0.025	0.956 ± 0.012	0.908 ± 0.040
	db2	0.876 ± 0.032	0.953 ± 0.016	0.940 ± 0.017	0.952 ± 0.019	0.955 ± 0.014	0.911 ± 0.031
	Haar	0.879 ± 0.027	0.955 ± 0.014	0.941 ± 0.015	0.951 ± 0.018	0.958 ± 0.016	0.910 ± 0.026
	bior1.3	0.874 ± 0.030	0.953 ± 0.015	0.939 ± 0.016	0.948 ± 0.020	0.958 ± 0.021	0.904 ± 0.027

## Data Availability

The data presented in this study are available on request from the corresponding author if data sharing is approved by ethics committee. The data are not publicly available due to data protection laws and conditions stated by the ethics committee.

## Supplementary Materials

The following are available online at https://www.mdpi.com/article/10.3390/cancers13081784/s1, Table S1: Legend of abbreviations and acronyms used in the research.
