# Supplementary material for: An Enhanced Histopathology Analysis: An AI-Based System for Multiclass Grading of Oral Squamous Cell Carcinoma and Segmenting of Epithelial and Stromal Tissue
Source: Cancers (Basel). 2021 Apr 8;13(8):1784. doi: 10.3390/cancers13081784 (PMC8068326; doi:10.3390/cancers13081784)
Supplement: Supplementary file 1 [file cancers-13-01784-s001.pdf]

Table S1. Legend of abbreviations and acronyms used in the research.

Abbreviation / acronyms	Explanation
OC	Oral Cancer
OSCC	Oral Squamous Cell Carcinoma
TNM	Tumor-Node-Metastasis
COE	Conventional Oral Examination
AI	Artificial Intelligence
CNN	Convolutional Neural Network
SVM	Support-Vector Machine
DAG	Directed Acyclic Graph
SWT	Stationary Wavelet Transform
HSI	Hyperspectral Imaging
MRI	Magnetic Resonance Imaging
MeMoSA	Mobile Mouth Screening Anywhere
ROI	Region of Interest
H&E	Hamatoxylin and Eosin
WSI	Whole Slide Image
WHO	World Health Organization
AJCC	American Joint Committee on Cancer
MT	Metallothionein
PBS	Phosphate-Buffered Saline
BSA	Bovine Serum Albumin
DAB	Diaminobenzidine
WT	Wavelet Transform
DWT	Discrete Wavelet Transform
L_D	Low pass filter
H_D	High pass filter
LL	Approximation coefficients
LH	Horizontal coefficients
HL	Vertical coefficients
HH	Diagonal coefficients
CM	Coefficient Mapping function
ILSVRC	ImageNet Large Scale Visual Recognition Challenge
ASPP	Atrous Spatial Pyramid Pooling
AUC	Area Under the Curve
mIOU	Mean Intersection-Over-Union
F1	Dice Coefficient
ACC	Accuracy
TPR	True Positive Rate
FPR	False Positive Rate
TP	True Positive
TN	True Negative
FN	False Negative
FP	False Positive
SGD	Stochastic Gradient Descent

HPC	High Performance Computing
TSR	Tumor-Stroma Ratio
TILs	Tumor-Infiltrating Lymphocytes
